# The Effects of Progressive Muscle Relaxation on Mental Health and Sleep Quality in Adults with Cystic Fibrosis: A Randomized Controlled Trial

**DOI:** 10.3390/jcm14082807

**Published:** 2025-04-18

**Authors:** Adelina Maritescu, Camelia Corina Pescaru, Alexandru Florian Crisan, Emil Robert Stoicescu, Cristian Oancea, Daniela Iacob

**Affiliations:** 1 Doctoral School, “Victor Babes” University of Medicine and Pharmacy Timisoara, 300041 Timisoara, Romania; adelina.maritescu@umft.ro (A.M.); stoicescu.emil@umft.ro (E.R.S.); 2 Pulmonary Rehabilitation Center, Clinical Hospital of Infectious Diseases and Pulmonology, “Victor Babes”, 300310 Timisoara, Romania; crisan@umft.ro; 3 Center for Research and Innovation in Personalized Medicine of Respiratory Diseases (CRIPMRD), “Victor Babes” University of Medicine and Pharmacy Timisoara, 300041 Timisoara, Romania; oancea@umft.ro; 4 Research Center for the Assessment of Human Motion, Functionality and Disability (CEMFD), “Victor Babes” University of Medicine and Pharmacy Timisoara, 300041 Timisoara, Romania; 5 Department of Radiology and Medical Imaging, ‘Victor Babes’ University of Medicine and Pharmacy Timisoara, 300041 Timisoara, Romania; 6 Research Center for Pharmaco-Toxicological Evaluations, ‘Victor Babes’ University of Medicine and Pharmacy Timisoara, 300041 Timisoara, Romania; iacob.daniela@umft.ro; 7 Pulmonology Clinic, Clinical Hospital of Infectious Diseases and Pulmonology, “Victor Babes”, 300310 Timisoara, Romania; 8 Department of Neonatology, ‘Victor Babes’ University of Medicine and Pharmacy Timisoara, 300041 Timisoara, Romania

**Keywords:** cystic fibrosis, mental health, progressive muscle relaxation

## Abstract

**Background/Objective**: Cystic fibrosis (CF) is a chronic genetic disease affecting multiple body systems and having a significant impact on mental health and sleep. Patients with CF frequently suffer from anxiety, depression, and sleep disturbances, but non-pharmacological strategies are understudied. Although progressive muscle relaxation (PMR) has recognized benefits, its impact on CF remains insufficiently explored. The study aimed to analyze the effect of integrating PMR into a standard pulmonary rehabilitation (PR) program on mental health, sleep quality, and quality of life in adults with CF. **Methods**: A total of 22 adult patients with CF were randomly assigned to either the intervention group (PR + PMR) or the control group (PR only). Assessments were performed at baseline, after 21 days of intervention, and at the 48-day follow-up. Outcome measures included the CFQ-R for quality of life, the HADS for mental health, and the PSQI for sleep. **Results**: Compared to the control group, participants who practiced PMR experienced significant reductions in anxiety (*p* = 0.05) and depression (*p* = 0.02) at the final assessment. A significant improvement in sleep quality was also observed (*p* < 0.01). No relevant differences were found in pulmonary function or performance on the six-minute walk test. **Conclusions**: Integrating PMR into pulmonary rehabilitation programs may be an effective strategy for improving mental health and sleep in patients with CF. Due to its accessibility and ease of implementation, PMR could be adopted as a complementary method in the holistic care of these patients.

## 1. Introduction

Cystic fibrosis (CF) is a chronic genetic disease that mainly affects the respiratory and digestive systems, significantly impacting patients’ quality of life. Although advances in pharmacological treatments and disease management have improved the prognosis, patients with CF continue to face challenges related to mental health and sleep quality [1]. The physical and psychological demands of living with CF frequently lead to significant mental health issues, particularly anxiety and depression [2].

Recent studies highlight a high prevalence of anxiety and depression symptoms among patients with cystic fibrosis, considerably affecting quality of life and adherence to treatments. A meta-analysis by Guta et al. reported an overall prevalence of anxiety of 29% and depression of 27% in patients with CF, highlighting a significant and often underdiagnosed psychological burden [3]. Regarding sleep disorders, research shows an increased incidence of insomnia, sleep fragmentation, and decreased sleep quality, influenced by nocturnal respiratory symptoms, chronic inflammation, and high-stress levels [4,5]. These disorders not only affect mental health but also aggravate the physical symptoms of the disease, creating a vicious cycle between respiratory status, psychological stress, and sleep quality. Thus, non-pharmacological interventions aimed at emotional regulation and relaxation, such as progressive muscle relaxation (PMR), become relevant within a holistic therapeutic approach.

Progressive muscle relaxation is a technique used to reduce sympathetic nervous system activation and improve emotional regulation [6]. It consists of successively contracting and relaxing muscle groups, with beneficial effects on symptoms of anxiety and depression and sleep quality [2]. Previous studies have demonstrated the effectiveness of PMR in reducing stress and improving sleep in patients with various chronic conditions [6]. Still, the impact of this technique on patients with CF remains underexplored. To our knowledge, this is the first study to specifically evaluate the effects of integrating PMR into a standard pulmonary rehabilitation program for adults with CF, with a focus on quality of life, mental health, and sleep quality. Given the high frequency of emotional and sleep disorders among these patients, identifying effective complementary methods, such as PMR, may contribute to optimizing rehabilitation programs and improving their care.

This study evaluates the effects of integrating progressive muscle relaxation (PMR) into a standard pulmonary rehabilitation program on quality of life, mental health, and sleep in adults with cystic fibrosis.

## 2. Materials and Methods

The reporting of this study conforms to the Consolidated Standards of Reporting Trials (CONSORT) statement.

### 2.1. Study Design

**Figure 1:**
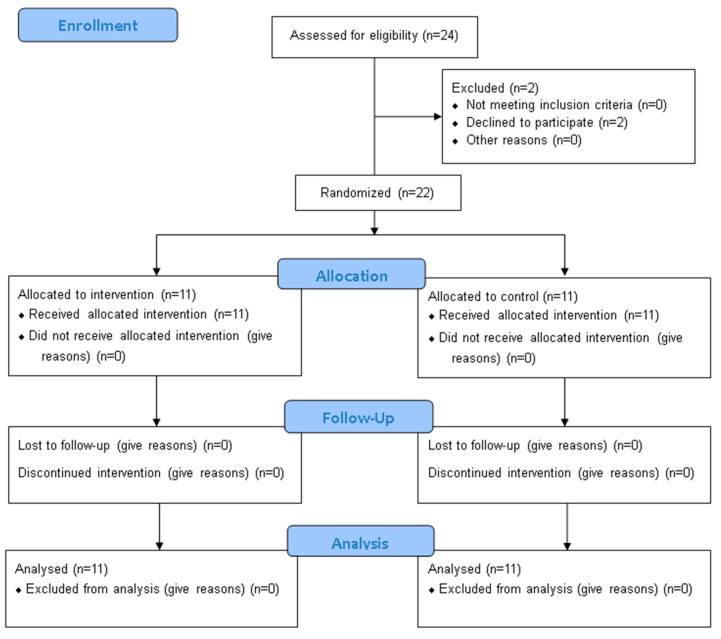
CONSORT 2010 Flow Diagram.

This study employed a randomized controlled trial (RCT) design to evaluate the effects of integrating PMR into a standard PR program on quality of life, mental health, sleep quality, and physical endurance in adults with CF (Figure 1). The study was conducted at the Pulmonary Rehabilitation Center, Clinical Hospital of Infectious Diseases and Pulmonology, "Victor Babeș” in Timisoara, Romania, between 2023 and 2024.

### 2.2. Participant Recruitment and Consent

CF patients were recruited from those seeking treatment at the Pulmonary Rehabilitation Center. This research study gained approval from the ethics committee of the Victor Babes Clinical Hospital (4989) and was registered on clinicaltrials.gov (NCT06592742) according to the requirements for reporting randomized controlled trials.

All of the participants were informed about the study objective and procedures. Before enrollment, written informed consent was obtained from each participant. The study protocol was approved by the local ethics committee and conducted in accordance with the Declaration of Helsinki [7].

Patients with CF were included in the study if they met the following criteria: confirmed diagnosis of CF based on clinical genetic testing or sweat chloride according to established CF diagnostic criteria; age 18 years or older at enrollment; stable lung function as defined by Forced Expiratory Volume in one second (FEV_1_); no pulmonary exacerbation requiring hospitalization or intravenous (IV) antibiotics in the four weeks before study entry, physical ability to participate in daily exercise, and components of lung clearance airway of the PR program; availability and ability to participate in daily PMR sessions; stable medication regimen for at least four weeks prior to study enrollment; non-smoker or quit smoking for at least six months before enrollment; ability to understand and provide written informed consent indicating willingness to participate; compliance with the intervention program and compliance with the follow-up assessments.

Patients were excluded if they met any of the following criteria: history of pulmonary exacerbation or acute respiratory infection requiring hospitalization or IV antibiotics in the past four weeks; history of lung transplantation or significant surgery in the past six months; severe comorbid conditions such as uncontrolled cardiovascular disease, renal or hepatic impairment, severe musculoskeletal disorders, severe psychiatric disorders, or uncontrolled significant changes in their medication regimen in the past four weeks; uncontrolled diabetes.

Based on the inclusion and exclusion criteria, we randomly assigned 22 patients into two groups using an online randomization tool. The intervention group consisted of 11 patients undergoing PR and PMR. The control group included 11 patients but followed only the PR program.

The primary outcomes of this study included anxiety and depression scores, assessed by the HADS, and sleep quality, measured by the PSQI. These were selected due to their clinical relevance in the context of cystic fibrosis, where emotional and sleep disturbances are common and significant for the patient’s quality of life.

Secondary outcomes included quality of life, assessed by the CFQ-R, lung function (FEV1, FVC, and FEV1/FVC ratio), and exercise capacity, measured by the 6MWT. These variables were monitored to explore possible additional effects of the intervention on general health and physical performance.

### 2.3. Data Collection

At admission, all of the participants underwent a pulmonary function test (PFT), a six-minute walk test (6MWT), completed a health-related quality of life questionnaire, the Cystic Fibrosis Questionnaire-Revised (CFQ-R), an instrument used to assess symptoms of anxiety and depression, the Hospital Anxiety and Depression Scale (HADS), and a sleep quality questionnaire, the Pittsburgh Sleep Quality Index (PSQI).

### 2.4. Outcome Measures

#### 2.4.1. Health-Related Quality of Life

The CFQ-R is a disease-specific tool designed to assess the health-related quality of life in individuals with CF. It includes 47 items that cover several of the following domains: physical functioning, emotional well-being, vitality, social functioning, body image, eating problems, treatment burden, respiratory symptoms, and digestive symptoms. Scores range from 0 to 100, with higher scores indicating better quality of life. The CFQ-R is used in clinical trials, research, and routine care to measure the impact of CF on daily life and the effectiveness of interventions [8].

#### 2.4.2. Mental Health

The HADS is a widely used tool to assess the symptoms of anxiety and depression in patients with physical health conditions. It consists of 14 items, divided into 2 subscales: 7 for anxiety (HADS-A) and 7 for depression (HADS-D). Each item is scored on a scale from 0 to 3, with total scores ranging from 0 to 21 for each subscale. Higher scores indicate greater levels of anxiety or depression. Scores are categorized as normal (0–7), mild (8–10), moderate (11–14), or severe (15–21) [9].

#### 2.4.3. Sleep Quality

The PSQI is a widely used tool designed to assess sleep quality and identify sleep disturbances over 1 month. It consists of 19 self-rated items grouped into the following 7 components: sleep duration, sleep latency, sleep efficiency, sleep disturbances, use of sleep medication, daytime dysfunction, and subjective sleep quality. Each component is scored from 0 to 3, with a total score ranging from 0 to 21; higher scores indicate worse sleep quality. A total score above 5 suggests poor sleep quality [10].

#### 2.4.4. Physical Endurance

Physical capacity was evaluated with the 6MWT, in which the patient had to walk as much as possible in the allotted time. To perform the 6MWT, we used an ERS/ATS (European Respiratory Society/American Thoracic Society) registration form, the BORG scale, a pulse oximeter, a blood pressure monitor, and a stopwatch. The test was performed according to ATS guidelines [11].

#### 2.4.5. Lung Function

We used the Smart PFT UI device Medical Equipment Europe GmbH provided to assess the pulmonary volumes. Spirometry is a non-invasive pulmonary function test that measures various lung volumes and flow rates during a forced expiration [12]. We evaluated the following parameters: the forced vital capacity (FVC), forced expiratory volume in the first second (FEV_1_), and FEV_1_/FVC ratio post-bronchodilator.

### 2.5. Intervention

#### 2.5.1. Pulmonary Rehabilitation Program

The PR program was structured according to the American Thoracic Society (ATS) guidelines [13] and designed using the FITT (Frequency, Intensity, Time, and Type) principle. The intervention was administered daily for 21 consecutive days under supervision. Each session included aerobic exercises, strength training with light weights, flexibility exercises, and breathing exercises. Education sessions on bronchial hygiene, postural drainage techniques, and pursed-lip breathing were also included. The intensity and duration of the exercises were individualized depending on the capacity of each patient, with a gradual progression over the 3 weeks, according to the protocol previously used in studies published by the authors [14].

#### 2.5.2. Progressive Muscle Relaxation Program

The PMR program is an essential non-pharmacological method, complementary to the RP program, with the main objectives of reducing anxiety, diminishing depression, and improving sleep quality [15]. The PMR program was applied daily in guided sessions held in the afternoon, lasting approximately 20–25 min in quiet spaces within the clinic under the guidance of a specialist. The protocol used was based on the Jacobson method and consisted of the controlled contraction and relaxation of the main muscle groups in the standardized order of head, shoulders, arms, chest, abdomen, and legs. The exercises were accompanied by verbal guidance on focusing on muscle sensations and training on conscious breathing. Patients were encouraged to practice the technique at home, but all of the sessions recorded in the study were supervised. The PMR protocol was taken over and adapted from a previous study by the same research team [14].

### 2.6. Statistical Analysis

All of the statistical analyses were conducted using MedCalc^®^ software version 22.016 (MedCalc Software Ltd, Ostend, Belgium; https://www.medcalc.org; 2023). software. Descriptive statistics were used to summarize the demographic and baseline characteristics of the study population. Data were presented as the mean ± standard deviation (SD) for continuous variables and as frequencies (percentages) for categorical variables.

An intention-to-treat approach was employed for the primary analysis to assess the differences between the intervention and control groups. The Shapiro–Wilk test was used to determine the normality of the data distribution. Depending on the data distribution, a paired *t*-test (for normally distributed data) or a Wilcoxon signed-rank test (for non-normally distributed data) was used to compare the within-group pre- and post-intervention differences. Between-group comparisons were performed using an independent *t*-test for normally distributed variables and the Mann–Whitney U test for non-normally distributed variables.

To evaluate changes in quality of life, mental health, sleep quality, and physical endurance, repeated measures analysis of variance (ANOVA) or Friedman tests were applied, as appropriate, to assess time effects and group interactions. Bonferroni post hoc analysis was performed for multiple comparisons where ANOVA was significant.

For categorical variables, such as classification categories in the HADS (anxiety and depression severity) and PSQI (poor vs. good sleep quality), the Chi-square test or Fisher’s exact test was used to analyze the differences between groups.

Effect sizes (Cohen’s d for *t*-tests, partial eta squared for ANOVA, and rank–biserial correlation for Mann–Whitney U tests) were calculated to assess the magnitude of differences. Additionally, Pearson or Spearman correlation coefficients were used to assess the relationships between outcome measures, such as changes in the FEV_1_ and quality of life scores. 

### 2.7. Sample Size

To assess the adequacy of the sample size, a post hoc analysis was conducted using the following two primary outcome measures with statistically significant results: the HADS-Depression score and the Pittsburgh Sleep Quality Index (PSQI), both evaluated at the final follow-up (T48). For the HADS-Depression score, the control group showed a median of 5.00 (IQR: 4.50–5.50), while the intervention group scored 3.50 (IQR: 3.00–4.25). The resulting effect size (Cohen’s d) was 2.01, indicating a very large effect. Based on this, only 5 participants per group would be needed to achieve 80% power, or 6 per group for 90% power (α = 0.05, two-tailed).

Similarly, for the PSQI score, the control group reported a mean of 5.55 ± 1.67, while the intervention group had 2.75 ± 1.30, yielding an effect size of 1.86. The minimum required sample size to detect this difference was 6 participants per group for 80% power and 7 per group for 90% power.

These findings confirm that the actual sample size of 11 participants per group was sufficient to detect clinically and statistically significant differences in both depression symptoms and sleep quality. All of the calculations were performed using the TTestIndPower function from the Statsmodels package in Python (version 0.14.0), applying a two-sample *t*-test approach.

## 3. Results

The study compared demographic, physiological, and quality of life parameters between the control and intervention groups at T0. No statistically significant differences were found between the two groups in any parameter (all *p*-values > 0.05). Both groups had an equal proportion of males (50%), and a similar median age (24 years), weight, height, BMI, and lung function (FVC, FEV_1_, and FEV_1_/FVC). Quality of life measures, including physical, emotional, social, and health-related scores and anxiety, depression, and sleep quality, showed no significant variations. Additionally, performance in the six-minute walk test and its predicted percentage were comparable. All of the findings at the start of the evaluation (T0) are presented in Table 1.

Table 2 presents the evolution of the CFQ-R questionnaire parameters at three timepoints (T0, T21, and T48) in the control and intervention groups. Across all domains, including physical, vitality, emotion, eating, treatment adherence, health perception, social functioning, body image, role, weight, respiratory, and digestive parameters, no statistically significant differences were observed between the groups at any timepoint. Both groups demonstrated relatively stable scores over time, with slight variations in some domains, such as improved emotional well-being and respiratory function at later timepoints. However, these changes did not reach statistical significance, suggesting that the intervention did not lead to notable differences in the CFQ-R scores compared to the control group.

**Figure 2:**
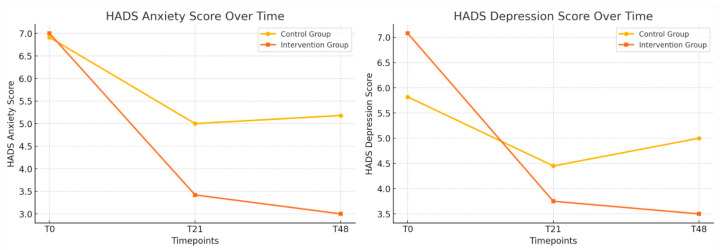
Longitudinal changes in anxiety and depression between the control and intervention groups; the evolution of the HADS-Anxiety and HADS-Depression scores (T0, T21, and T48) in the control and intervention groups.

**Figure 3:**
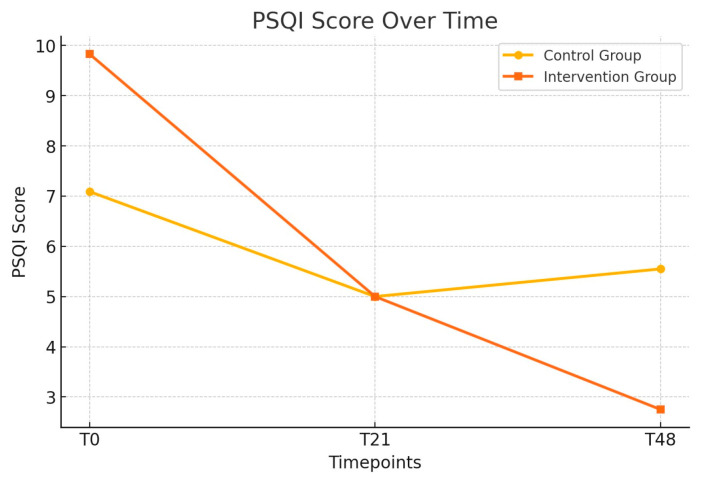
Longitudinal changes in the PSQI scores between the control and intervention groups; the evolution of the PSQI scores over time (T0, T21, and T48) in the control and intervention groups.

Table 3 presents the longitudinal changes in anxiety, depression, sleep quality (PSQI), and exercise capacity (6MWD) between the control and intervention groups. Anxiety scores (HADS-Anxiety) showed a trend toward improvement in the intervention group, reaching borderline significance at T48 (*p* = 0.05). Depression scores (HADS-Depression) significantly improved in the intervention group at T48 (*p* = 0.02), and the total HADS score also demonstrated a significant reduction at this timepoint (*p* = 0.01) (Figure 2). Sleep quality showed significant improvement in the intervention group at T48 (*p* < 0.01). These improvements are presented in Figure 3. However, no significant differences were observed in the 6MWD or its percentage from the predicted values at any time.

To provide a more precise estimate of the effect of the intervention, mean differences with 95% confidence intervals (CIs) were calculated for the key outcomes at the 48-day follow-up (T48). For the HADS-Anxiety score, the mean difference between the control and intervention groups was 2.18 points (95% CI: 0.16 to 4.20), indicating a moderate and clinically relevant reduction in anxiety symptoms. The HADS total score showed a mean difference of 3.95 points (95% CI: 1.29 to 6.61), supporting the positive global impact of PMR on mental health. Sleep quality, as measured by the PSQI, improved significantly in the intervention group, with a mean difference of 2.80 points (95% CI: 1.47 to 4.13). Regarding physical endurance, the mean difference in the six-minute walk distance (6MWD) was −25.00 m (95% CI: −83.37 to 33.37), and, for the percentage of the predicted 6MWD, the difference was 0.00% (95% CI: −12.48 to 12.48), indicating no significant between-group effects in functional capacity.

To assess the magnitude of the observed effects, Cohen’s d was calculated for key outcomes at the 48-day follow-up (T48). The effect size for the HADS-Depression score was 2.01, indicating a very large reduction in depressive symptoms among patients who received progressive muscle relaxation (PMR) in addition to pulmonary rehabilitation. Similarly, the effect size for sleep quality, measured by the PSQI, was 1.86, also reflecting a very large effect. These values provide strong support for the clinical relevance of the intervention, particularly in improving psychological well-being and sleep among adults with cystic fibrosis. According to established interpretation guidelines, values above 0.80 represent large effects, and those exceeding 1.2 suggest very large effects. Therefore, the improvements observed in the intervention group are not only statistically significant but also of meaningful, practical importance.

## 4. Discussion

The aim of this study was to evaluate the effects of incorporating PMR into a standard pulmonary rehabilitation program, with a focus on its potential benefits for mental health, sleep quality, and overall quality of life in adult patients diagnosed with CF.

In patients with CF, chronic physiological stress generated by persistent inflammation, hypoxia, and recurrent respiratory infections can lead to excessive activation of the sympathetic nervous system and the hypothalamic–pituitary–adrenal axis, which is associated with increased cortisol levels and reduced emotional regulation capacity [16]. This imbalance contributes to the frequent occurrence of symptoms of anxiety, depression, and sleep disorders among patients with CF [5]. In this context, techniques such as PMR can play an essential role. By controlling the contraction and relaxation of muscle groups, PMR reduces sympathetic activation, favoring parasympathetic dominance and inducing a physiological relaxation response [5]. Furthermore, from a psychological perspective, this technique offers patients an active strategy for emotional self-regulation, reducing cognitive rumination and improving the perception of control over their own symptoms [17]. Thus, integrating PMR into pulmonary rehabilitation programs can contribute not only to the improvement of psychological symptoms but also to the overall improvement of quality of life in CF.

The results show a significant reduction in anxiety and depression symptoms in the intervention group, with lower HADS scores at the end of the study. These data suggest that PMR may be an effective tool for emotional regulation. The literature supports these findings, which have previously demonstrated the beneficial effects of relaxation techniques on reducing sympathetic nervous system activity and improving stress response [18].

In addition to its physiological effects, PMR may have a significant impact on the cognitive and emotional processing of cystic fibrosis symptoms. People with chronic diseases, including CF, frequently develop coping mechanisms to deal with daily symptoms. Previous studies suggest that relaxation-based strategies can improve emotional regulation by reducing rumination and excessive stress responses [19]. PMR provides patients with an active tool for managing physical and psychological discomfort, which may explain the reduction in anxiety and depression observed in our study.

An important aspect of this study was the assessment of the quality of life using the CFQ-R, a validated and widely used instrument to measure the impact of CF on general health status [20]. It includes several relevant domains, such as physical functioning, vitality, emotional well-being, body image, and respiratory symptoms. In our study, the most significant improvements were observed in vitality and emotional well-being domains, indicating that integrating PMR may enhance the perception of daily energy and overall emotional well-being. These results are consistent with previous studies showing that relaxation techniques improve the subjective perception of health and reduce the negative impact of chronic symptoms on quality of life [6,21].

However, other domains, such as body image and respiratory symptoms, did not significantly improve. This can be explained by the fact that PMR has a more significant impact on psychological components and a lesser effect on objective physical symptoms, which are significantly influenced by specific medical treatments and the severity of the disease [22]. It is also possible that the duration of the intervention was insufficient to generate significant changes in these aspects of quality of life.

Patients who practiced PMR showed a significant improvement in PSQI scores (*p* < 0.01) regarding sleep quality, confirming the effectiveness of this technique in alleviating sleep disorders. This finding aligns with previous studies that highlighted the benefits of relaxation methods in enhancing sleep architecture for patients with chronic respiratory diseases [23]. It is well documented that patients with CF are at increased risk of sleep disturbances, including insomnia and fragmented sleep, caused by nocturnal respiratory symptoms and high stress levels. Previous studies suggest that relaxation techniques can improve sleep architecture by reducing sleep latency and increasing the duration of deep sleep. This is particularly important for patients with chronic conditions, as quality sleep plays a critical role in physiological recovery and strengthening immunological defense mechanisms [24]. Improving sleep is essential for CF patients, as it can positively influence physical and mental health outcomes. While the 6MWT test did not reveal significant differences between groups, the observed trend of increased distance traveled in the PMR group indicates a potential advantage for physical endurance. This improvement may be attributed to the progressive nature of PR, which improves muscle strength and cardiovascular fitness. In addition, PMR may have indirectly supported physical performance by reducing perceived fatigue and anxiety, as reflected in lower Borg fatigue scores.

The improvements observed in mental health and sleep quality can be explained by the ability of PMR to modulate the activity of the autonomic nervous system, reducing sympathetic hyperactivation and favoring parasympathetic predominance. Relaxation techniques, such as PMR, have also been associated with the stimulation of neuroplasticity, which may contribute to long-term benefits in terms of symptoms of anxiety and depression [24]. These physiological mechanisms support the role of PMR as a complementary therapeutic tool in pulmonary rehabilitation programs. Given the positive effects of PMR on quality of life, mental health, and sleep, integrating this technique into standard pulmonary rehabilitation programs could enhance the patient-centered approach [6]. A significant advantage of PMR is its accessibility—it requires minimal resources, can be practiced at home, and does not interfere with pharmacological treatments. Healthcare professionals should consider including relaxation techniques in cystic fibrosis management plans, providing structured guidance for their application and maintenance in the long term. One of the main limitations of this study is the relatively small sample size, which may reduce the generalizability of the findings. However, this limitation must be interpreted in the context of the local epidemiology of cystic fibrosis. In the western region of Romania, only 24 adult patients with cystic fibrosis are currently under follow-up across the neighboring counties. This severely restricts the potential recruitment pool for interventional studies. The use of parametric statistical tests, although supported by the verification of data normality, does not provide maximum certainty on the Gaussian distribution of the variables, precisely because of the small number of participants. This aspect may affect the robustness of the results and the interpretation of the effect sizes. Also, the study design did not allow for multivariate analyses by time and group, which would have provided a more detailed perspective on the interactions between the variables and their evolution over time.

There is a lack of statistically significant results in some areas of the CFQ-R questionnaire. Although the trend is favorable, the significant standard deviation may indicate high individual variability, influencing the statistical significance of the observed improvements. Second, the duration of the intervention may not have been sufficient to produce significant changes in all domains of quality of life. Furthermore, the effects of PMR on objective lung function parameters were unimportant, indicating that this technique has a more substantial impact on subjective aspects of well-being. Future research should involve larger samples and explore the long-term effects of incorporating PMR in the rehabilitation of cystic fibrosis patients.

The lack of a long-term follow-up period limits the conclusions regarding the maintenance of the observed effects over time. These aspects should be taken into account both in the interpretation of the current results and in the planning of future research.

Another aspect to consider is that, at baseline (T0), the depression and sleep quality (PSQI) scores were slightly higher in the intervention group compared to the control group, although the differences were not statistically significant. This raises the hypothesis that patients with higher levels of psychological distress and sleep disturbances might benefit more from relaxation techniques such as PMR. It is possible that more severe symptomatology at baseline provides a greater “room” for improvement, and the effects of the intervention may thus be easier to observe. In this regard, interventions such as PMR might be particularly useful in subgroups of patients identified as being at high risk for anxiety, depression, or sleep disturbances. Further research is needed to assess whether the initial level of emotional distress or sleep may be a predictive factor of the effectiveness of PMR, thus contributing to a more personalized approach to rehabilitation.

## 5. Conclusions

Integrating PMR into the PR program resulted in significant reductions in anxiety and depression, as well as improved sleep quality in patients with CF. No relevant changes in lung function or performance on the six-minute walk test were observed. PMR may be used as an effective complementary intervention to optimize mental health and sleep in patients with cystic fibrosis. However, these results should be interpreted with caution, given the small sample size, the use of parametric statistics on a small group, and the inability to apply multivariate analysis. Further research with larger samples is needed to validate these results and assess long-term effects.

## Figures and Tables

**Table 1:** Baseline characteristics and outcome measures of the control and intervention groups at T0.

Parameters	Control Group	Intervention Group	*p*-Value
Male gender	6 (54.54%)	6 (50%)	0.83
Age (years)	24.00 (19.50–25.00)	24.00 (20.00–29.00)	0.40
Weight (kg)	56.00 ± 7.95	51.75 ± 11.02	0.32
Height (m)	1.67 (1.63–1.78)	1.67 (1.62–1.74)	0.85
BMI	19.45 ± 1.84	18.25 ± 2.76	0.25
FVC (L)	3.04 ± 0.98	3.27 ± 1.33	0.65
FVC (%)	71.00 ± 22.21	74.42 ± 27.33	0.76
FEV_1_ (L)	2.15 ± 0.87	2.34 ± 1.19	0.67
FEV_1_ (%)	58.36 ± 24.63	61.50 ± 28.38	0.79
FEV_1_/FVC (%)	77.45 ± 14.04	81.17 ± 15.53	0.57
Physical	49.65 ± 31.53	59.01 ± 36.24	0.53
Vitality	41.60 (17.15–75.00)	49.95 (22.90–93.70)	0.62
Emotion	66.55 ± 28.05	63.87 ± 29.00	0.83
Eating	100.00 (72.25–100.00)	83.25 (61.05–100.00)	0.38
Treat	80.00 (77.20–89.35)	88.80 (49.95–91.60)	0.95
Health	46.43 ± 27.62	57.37 ± 30.37	0.40
Social	54.88 ± 32.04	52.74 ± 27.27	0.87
Body	50.23 ± 18.48	47.82 ± 20.05	0.77
Role	67.60 (58.15–72.00)	66.60 (58.30–77.08)	0.69
Weight	100.00 (100.00–100.00)	100.00 (100.00–100.00)	0.64
Respiratory	44.40 (36.15–83.40)	41.60 (27.70–88.80)	0.62
Digestive	88.80 (81.15–100.00)	100.00 (72.15–100.00)	0.82
Anxiety	6.91 ± 3.00	7.00 ± 2.61	0.94
Depression	5.82 ± 2.69	7.08 ± 2.47	0.28
PSQI	8.09 ± 1.81	9.83 ± 3.02	0.12
6MWD (m)	570.00 (510.00–635.00)	570.00 (513.75–631.25)	0.88
6MWD (% pred)	81.00 (70.50–92.50)	83.50 (72.25–91.50)	0.98

The data are expressed as arithmetic mean ± SD/median and [IQR] according to the Shapiro–Wilk test. BMI—body mass index; FVC—forced vital capacity; FEV_1_—forced expiratory volume; PSQI—Pittsburgh Sleep Quality Index; 6MWD—six-minute walk distance.

**Table 2:** Longitudinal comparison of the CFQ-R questionnaire parameters between the control and intervention groups.

CFQ-R Questionnaire Parameter	Timepoint	Control Group	Intervention Group	*p*-Value
Physical	T0	49.65 ± 31.53	59.01 ± 36.24	0.53
	T21	50.49 ± 33.29	54.14 ± 30.72	0.80
	T48	49.68 ± 33.29	54.23 ± 30.73	0.75
Vitality	T0	41.60 (17.15–75.00)	49.95 (22.90–93.70)	0.62
	T21	50.65 ± 30.17	50.65 ± 28.96	1.00
	T48	50.09 ± 30.09	50.86 ± 29.01	0.95
Emotion	T0	66.55 ± 28.05	63.87 ± 29.00	0.83
	T21	80.20 (25.12–99.15)	89.95 (55.00–100.00)	0.92
	T48	86.60 (34.50–97.50)	89.80 (55.00–99.25)	0.69
Eating	T0	100.00 (72.25–100.00)	83.25 (61.05–100.00)	0.38
	T21	100.00 (82.70–100.00)	100.00 (86.03–100.00)	0.92
	T48	95.50 (82.45–99.00)	100.00 (86.10–100.00)	0.27
Treat	T0	80.00 (77.20–89.35)	88.80 (49.95–91.60)	0.95
	T21	100.00 (59.65–100.00)	72.15 (47.17–80.50)	0.09
	T48	90.00 (59.25–99.00)	72.15 (47.25–80.72)	0.19
Health	T0	46.43 ± 27.62	57.37 ± 30.37	0.40
	T21	47.07 ± 27.31	55.51 ± 30.41	0.51
	T48	46.68 ± 27.09	55.53 ± 30.40	0.49
Social	T0	54.88 ± 32.04	52.74 ± 27.27	0.87
	T21	55.65 ± 29.08	59.67 ± 26.35	0.74
	T48	55.45 ± 29.10	60.09 ± 25.43	0.70
Body	T0	50.23 ± 18.48	47.82 ± 20.05	0.77
	T21	63.11 ± 25.32	49.03 ± 30.23	0.26
	T48	62.65 ± 25.08	49.53 ± 29.45	0.28
Role	T0	67.60 (58.15–72.00)	66.60 (58.30–77.08)	0.69
	T21	75.50 (58.30–84.65)	79.15 (66.60–91.60)	0.69
	T48	75.00 (58.00–84.00)	79.65 (66.60–91.60)	0.54
Weight	T0	100.00 (100.00–100.00)	100.00 (100.00–100.00)	0.64
	T21	100.00 (100.00–100.00)	100.00 (100.00–100.00)	0.64
	T48	100.00 (100.00–100.00)	100.00 (100.00–100.00)	1.00
Respiratory	T0	44.40 (36.15–83.40)	41.60 (27.70–88.80)	0.62
	T21	58.10 ± 30.06	67.08 ± 25.53	0.47
	T48	57.95 ± 29.41	67.08 ± 25.53	0.46
Digestive	T0	88.80 (81.15–100.00)	100.00 (72.15–100.00)	0.82
	T21	100.00 (92.60–100.00)	100.00 (86.03–100.00)	0.78
	T48	95.00 (87.00–99.35)	100.00 (86.03–100.00)	0.48

The data are expressed as arithmetic mean ± SD/median and [IQR] according to the Shapiro-Wilk test.

**Table 3:** Longitudinal assessment of anxiety, depression, sleep quality, and exercise capacity in the control and intervention groups.

Parameters	Timepoint	Control Group	Intervention Group	*p*-Value
HADS-Anxiety	T0	6.91 ± 3.00	7.00 ± 2.61	0.94
	T21	5.00 ± 2.89	3.42 ± 1.61	0.15
	T48	5.18 ± 2.85	3.00 ± 1.47	0.05
HADS-Depression	T0	5.82 ± 2.69	7.08 ± 2.47	0.28
	T21	4.45 ± 2.31	3.75 ± 1.30	0.41
	T48	5.00 (4.50–5.50)	3.50 (3.00–4.25)	0.02
HADSTotal	T0	12.73 ± 4.69	14.08 ± 3.45	0.46
	T21	9.45 ± 4.05	7.17 ± 2.51	0.14
	T48	10.45 ± 3.73	6.50 ± 1.98	0.01
PSQI	T0	7.09 ± 2.07	9.83 ± 3.02	0.02
	T21	5.00 (3.50–5.50)	5.00 (4.00–7.25)	0.33
	T48	5.55 ± 1.67	2.75 ± 1.30	<0.01
6MWD (m)	T0	570.00 (510.00–635.00)	570.00 (513.75–631.25)	0.88
	T21	620.00 (583.75–670.00)	660.00 (580.00–685.00)	0.80
	T48	615.00 (501.25–632.50)	640.00 (600.00–665.00)	0.13
6MWD (%) from Predicted	T0	81.00 (70.50–92.50)	83.50 (72.25–91.50)	0.98
	T21	90.00 (85.87–99.00)	93.50 (85.00–100.00)	0.82
	T48	80.00 (66.00–86.50)	80.00 (79.00–92.00)	0.18

The data are expressed as arithmetic mean ± SD/median and [IQR] according to the Shapiro–Wilk test. HADS—Hospital Anxiety and Depression Scale; PSQI—Pittsburg Sleep Quality Index; 6MWD—six-minute walk distance.

## Data Availability

The supporting data for the findings of this study can be obtained by contacting the corresponding author upon request. However, the data cannot be publicly accessed due to privacy and ethical considerations.

## Abbreviations

The following abbreviations are used in this manuscript:

CF	Cystic Fibrosis
PR	Pulmonary Rehabilitation
PMR	Progressive Muscle Relaxation
CONSORT	Consolidated Standards of Reporting Trials
RCT	Randomized Controlled Trial
FEV_1_	Forced Expiratory Volume in one second
IV	Intravenous
PFT	Pulmonary Function Test
6MWT	Six Minute Walk Test
CFQ-R	Cystic Fibrosis Questionnaire-Revised
HADs	Hospital Anxiety and Depression Scale
PSQI	Pittsburgh Sleep Quality Index
ERS	European Respiratory Society
ATS	American Thoracic Society
FVC	Forced Vital Capacity
